# Pre and post-natal risk and determination of factors for child obesity

**Published:** 2016

**Authors:** LM Trandafir, OR Temneanu

**Affiliations:** * Mother and Child Department, “Grigore T. Popa” University of Medicine and Pharmacy, Iasi, Romania

**Keywords:** childhood obesity, obesity gene, lifestyle, unbalanced diet

## Abstract

Obesity is considered a condition presenting a complex, multi-factorial etiology that implies genetic and non-genetic factors. The way the available information should be efficiently and strategically used in the obesity and overweight prohylaxisprogrammes for children all over the world is still unclear for most of the risk factors.

Mothers’ pre-conception weight and weight gain during pregnancy are two of the most important prenatal determinants of childhood obesity. Maternal obesity and gestational weight gain are associated with foetal macrosomia and childhood obesity, and this effect extends into adulthood. Obesity and the metabolic syndrome in children originate in intrauterine life.

The current obesity epidemic is probably the result of our evolutive inheritance associated with the consumption of highly processed food with an increased calorific value. The determination of risk factors involved in child obesity are: genetic predisposition, diet, sedentary behaviors, socioeconomic position, ethnic origin, microbiota, iatrogenic, endocrine diseases, congenital and acquired hypothalamic defects, usage of medications affecting appetite.

However, the vast majority of patients will not have any of these identifiable conditions. Regardless of the aetiology, all the patients should be considered for modifiable lifestyle risk factors and screened for the complications of obesity.

## Introduction

The epidemic of obesity we are witnessing today is a consequence of the natural biological and technological evolution of humankind. According to the hypothesis postulated since the 1960s, the human species has adapted to the cyclic periods with lack of food (for example the ice age, the economic crises), which led to a natural selection of the individuals with an economical energetic metabolism, known as “the thrifty genotype hypothesis” [**1**].

The current obesity epidemic is probably the result of our evolutive inheritance associated with the consumption of highly processed food with an increased calorific value. One proof for this are the populations of North America, which have kept their traditional life style with daily physical exercise, in their case, the prevalence of obesity being considerably lower [**2**].

Even though the appearance of agriculture 14000 years ago ensured a more and more varied nourishment (but with a reduced calorific value), nevertheless, the daily activities required a high calorific and energetic value, until approximately 50 years ago, when radical changes in the food industry considerably improved the bio-availability of food and its energetic value. Lately, we have witnessed an increased prevalence of obesity among the developed countries as well as in the developing or less developed countries. However, in the less developed countries, the obesity epidemic is present mostly in urban areas, easily giving access to food, which is highly energetic and affordable [**3**].

Obesity in children teeangers represents a significant forecaster of obesity in adulthood and a risk factor for the numerous complications it generates. Thus, 20% of the obese newly born will become obese children, 40% of the obese children will become obese teenagers and 80% of the obese teenagers will become obese adults.

Nowadays, obesity is considered a condition presenting a complex, multi-factorial etiology that implies genetic and non-genetic factors. The way the available information should be efficiently and strategically used in the obesity and overweight prohylaxisprogrammes for children all over the world is still unclear for most of the risk factors. 

**Fig. 1:**
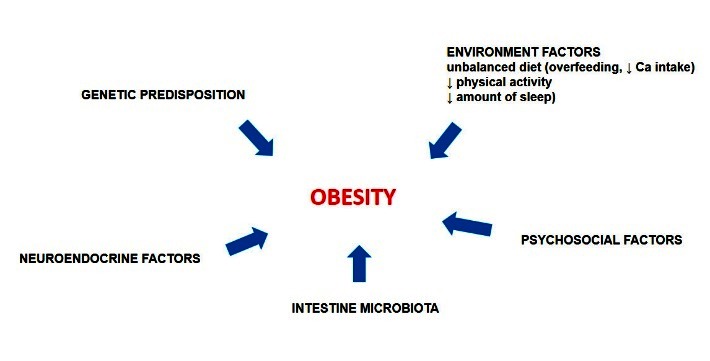
Multi-factor etiology of obesity

Obesity develops primarily because energy intake exceeds energy expenditure, and many environmental and host factors interact in complex ways to contribute to its development [**4**]. Individual behaviors, environmental factors and genetics all contribute to the complexity of the obesity epidemic (**Fig. 1**). 

## The determination of risk factors involved in child obesity 

**Genetic predisposition**

In obesity cases, only 20-50% of the variation in the body weight is influenced by the environment factors, while 50-80% is explained by genetic changes [**5**]. 

The most frequent forms of obesity are the polygenic ones. Several genes contribute to weight gain by controlling appetite, energy expenditure, and metabolism, but can only partially account for the development of obesity. There are currently more than 600 genes, markers and chromosomal regions known to be associated to Ob, the obesity gene. The obesity gene was discovered in 1994. This gene codifies a protein that is synthesized by adipocytes and is called leptin. Leptin acts as a hormone playing multiple roles in the body and its serum level is correlated to the fat quantity, varying depending on age and gender. Leptin acts at the level of the hypothalamus and it is involved in regulating the energy balance [**6**]. 

The monogenic determinism of obesity is rarely seen and it is characterised by the deficit of leptin, pro-opiomelanocrotin and leptin receptors [**7**]. Mutations in the gene encoding leptin (LEP) typically lead to an absence of circulating leptin and to extreme obesity. Otherepigenetic biomarkers of obesity are represented by FGF2, PTEN, CDKN1A and ESR1 implicated in adipogenesis, SOCS1/SOCS3, in inflammation, and COX7A1 LPL, CAV1, and IGFBP3, in intermediate metabolism and insulin signalling [**8**]. 

**Epigenetics**

The mechanism whereby in utero factors can produce heritable changes in adiposity has been suggested to be due to the DNA methylation or histone modification of DNA in gene regulatory regions. The environmental factors and maternal lifestyles, particularly adverse nutritional disturbances, proceed in early life to drive the risks for the onset of metabolic diseases and excessive weight gain in later life stages [**9**]. The maternal nutrition can program gene expression patterns to the embryo that persist into adulthood and may contribute to the appearance of hypertension, insulin resistance, hyperlipemia, and abdominal obesity. The parental conditions and lifestyles, which may involve maternal size/obesity, the use of nutritional supplements, alcohol or drug abuse, as well the administration of therapeutical agents in this critical period, may alter specific processes, with an impact on embryonic, placental and foetal growth, organogenesis or regulatory set points for system functions affecting adiposity, in which inflammatory and immunologically mediated processes may be involved [**10**]. 

Mothers’ pre-conception weight and weight gain during pregnancy are two of the most important prenatal determinants of childhood obesity. Several factors may influence the association of maternal weight and weight gain during pregnancy with long-term child health outcomes. These factors include maternal and paternal BMI, maternal smoking during pregnancy, blood sugar levels during pregnancy, fetal growth, birth weight, and infant feeding practices [**11**]. 

Maternal obesity and gestational weight gain are associated with foetal macrosomia and childhood obesity, and this effect extends into adulthood. In turn, childhood obesity increases chances of later life obesity, thus type 2 diabetes, and cardiovascular disease in the offspring [**12**]. 

The exposure to gestational diabetes is associated with an increased risk of childhood and early adult obesity in the offspring. Mothers with obesity or gestational diabetes mellitus have low circulating levels of adiponectin. Irving L et al. showed that adiponectin supplementation in pregnancy prevents fetal overgrowth caused by maternal obesity. Moreover, the authors demonstrated that adiponectin functions as an endocrine link between maternal adipose tissue and fetal growth by regulating the placental function. Therefore, the improvement of adiponectin levels in women with obesity and/or gestational diabetes may serve as an effective intervention strategy in preventing the intrauterine transmission of obesity and metabolic disease [**13**].

Recent findings suggested that a very low birth weight and a very high birth weight are both associated with childhood obesity. Although the link between very high birth weight and childhood obesity is studied more, the link between low birth weight and obesity may be the result of accelerated growth immediately after birth. Babies who were “deprived of nutrition” before birth may be primed for an accelerated growth after birth when exposed to a rich nutrient environment (which often consists of infant formula) [**14**]. This rapid growth in the first few months and perhaps even the first days of postnatal life, are associated with an increased risk of children being overweight.

An early age at BMI rebound is associated with a greater risk of obesity, but this may be a statistical artifact.

**Diet**

In addition to fetal “over-nutrition” or “under-nutrition”, it is possible that the developmental exposure to the endocrine disrupting chemicals (EDCs) or to other chemicals plays a role in the development of diabetes and childhood obesity. Some scientists have coined the term “obesogens” for chemicals that they believe may promote weight gain and obesity. Such chemicals may promote obesity by increasing the number of fat cells, changing the amount of calories burned at rest, altering energy balance, and altering the body’s mechanisms for appetite and satiety. Fetal and infant exposure to such chemicals may result in more weight gain per food consumed and possiblyless weight loss per amount of energy expended. The health effects of these chemicals during fetal and infant development may persist throughout life, long after the exposures occur [**15**].

It is likely that the protective effect of breastfeeding results from a combination of factors. First, the infant formula contains nearly twice as much protein per serving as breast milk. This excess protein may stimulate insulin secretion in an unhealthy way [**16**].

Second, the biological response to breast milk differs from that of the formula. When feeding a baby, the mother’s milk prompts the baby’s liver to release a protein that helps regulating metabolism [**17**]. Instead of breast milk,the feeding formula increases the baby’s concentrations of insulin in his or her blood, prolonging the insulin response [**18**] and, even in childhood, it is associated with unfavorable concentrations of leptin, a hormone that inhibits appetite and controls body fatness. Numerous barriers make breastfeeding difficult despite the well-known health benefits of breastfeeding and the preference of most pregnant women to breastfeed [**19**].

Parents and caregivers of babies may also benefit from guidance regarding the moment to start feeding them solid foods, since the early introduction of solids (prior to six months) increases the risk for childhood obesity [**20**]. Also, hypercaloric, hyperglucidic, hyperproteic nutrition, poor in fibres during the critical periods of childhood are involved in the increase of the risk of obesity. Weight gain usually involves the combination of consuming too many calories and not expending enough through physical activity. 

A high-energy intake in early infancy and a high consumption of sweetened drinks in childhood is prospectively associated with childhood obesity risk. The consumption of sugar-sweetened beverages, particularly carbonated soft drinks, may be a key contributor to the epidemic of overweight and obesity, by virtue of these beverages- high-added sugar content, low satiety, and incomplete compensation for total energy. WHO has developed guidance on free sugars intake, based on the impact of free sugars intake on weight gain and dental caries. The current evidence suggests that the increase in the consumption of sugar-sweetened beverages is associated with overweight and obesity in children. Free sugars include monosaccharides and disaccharides added to foods and beverages by the manufacturer, cook, or consumer, and sugars naturally present in honey, syrups, fruit juices and fruit juice concentrates [**21**].In 1977–78, children aged 6–11 drank about four times as much milk as soda. In 2001–02, they drank about the same amounts of milk and soda [**22**].

Researchers have hypothesized that TV watching could promote obesity in several ways: displacing time for physical activity; promoting poor diets; giving more opportunities for unhealthy snacking (during TV viewing); and even by interfering with sleep [**23**]. The American Academy of Pediatrics (AAP) recommends that children of two years old and under should not be exposed to television, and children over the age of two should limit daily media exposure to only 1-2 hours of quality programming [**24**].

Studies showed an association between television viewing and the risk of being overweight in preschool children, independent of socio-demographic factors. Specifically, for each additional hour of television viewing, the odds ratio of children having a BMI greater than the 85th percentile was 1.06. Children who have TV sets in their bedrooms are also more likely to gain excess weight than children who do not [**25**]. Television viewing is also linked to dietary intake. Studies have shown that the time spent in front of the TV is proportional to the increased ingestion of food, especially fast food [**26**]. 

**Sedentary behaviors**

Other sedentary behaviors—computer/Internet use, video game playing, sitting at work/school, driving, have not been studied as extensively as TV watching. However, there is evidence that these other forms of “sit time” can contribute to obesity. There is evidence that spending too much time sitting—at work or at home—increases the risk of becoming obese and may increase the risk of chronic diseases and early death [**27**]. It is necessary to decrease the time spent in sedentary behaviors, such as leisure-time Internet and computer use, in order to reduce the prevalence of overweight and obesity. Longitudinal studies are required to further examine the potential causal relationships between the development of overweight and the specific sedentary behaviors such as Internet and computer use [**28**].

For children and young people, physical activity includes playing, games, sports, transportation, chores, recreation, physical education, or planned exercise, in the context of family, school, and community activities. In order to improve cardiorespiratory and muscular fitness, bone health, and cardiovascular and metabolic health biomarkers, the following activities are required:

• Children and youth aged 5–17 should accumulate at least 60 minutes of moderate- to vigorous-intensity physical daily activity.

• Amounts of physical activity of more than 60 minutes provide additional health benefits.

• Most of the daily physical activity should be aerobic. Vigorous-intensity activities should be incorporated, including those that strengthen muscle and bone at least 3 times per week [**29**].

There is still a need for the research to determine the appropriate dose of exercise in combination with sedentary behaviours and other activities in the context of our modern lifestyle in order to prevent obesity at all ages [**30**]. It has also been suggested that physically active young people more readily adopt other healthy behaviours (e.g. avoidance of tobacco, alcohol, and drug use) and demonstrate higher academic performance at school.

Sleep is the “most sedentary activity”, yet may be the only one that protects from weight gain [**31**]. Sleep is an important modulator of neuroendocrine function and glucose metabolism; sleep loss has been shown to result in metabolic and endocrine alterations, including decreased glucose tolerance, decreased insulin sensitivity, increased evening concentrations of cortisol, increased levels of ghrelin, decreased levels of leptin, and increased hunger and appetite. Laboratory studies and multiple epidemiological studies have linked short-sleep duration and poor-sleep quality to obesity risk [**32**].Children who get less sleep in infancy and early childhood may be at greater risk of being overweight or obese during mid-childhood.

**Socioeconomic position**

In high-income countries, generations born prior to 1950’s–1960 did not show socioeconomic differentials in adiposity/obesity in childhood (as they did as adults). Children from low and middle-income countries tend to be stunted and underweight but, with sufficient nutrition, gain healthy weight and with overnutrition, are prone to obesity. Some evidence showed that contemporary populations of children present higher rates of obesity in those from the lowest socioeconomic groups in the high-income countries. Children living in low-income neighborhoods are 20 percent to 60 percent more likely to be obese or overweight than those living in high socioeconomic status neighborhoods and healthier built environments.

Children in urban areas are more likely to be obese than those in rural areas in many countries including high and low-middle income countries.

**Ethnic origin**

Racial and ethnic inequities also persistamong children; 22.5 percent of the Latino children and 20.2 percent of the black children are obese, compared to 14.1 percent of the non-Latino White and 6.8 percent of the Asian-American children.

**Microbiota**

The link between the microbes in the human gut and the development of obesity, cardiovascular disease, and metabolic syndromes, such as type 2 diabetes, is becoming clearer. The microbiota participatesinthe development and maintenance of the obese state, including host ingestive behavior, energy harvest, energy expenditure, and fat storage. The proposed mechanisms for the role of gut microbiota include the provision of additional energy by the conversion of dietary fiber to short-chain fatty acids, effects on gut-hormone production, and increased intestinal permeability causing elevated systemic levels of LPS. This metabolic endotoxemia is suggested to contribute to low-grade inflammation, a characteristic trait of obesity, and the metabolic syndrome. Finally, the activation of the endocannabinoid system by lipopolysaccharidesand/or high-fat diets is discussed as another causal factor [**33**].

**Influence of external factors on intestinal microbiota of infants**

The development of the intestinal microbiota in infants is characterized by rapid and large changes in microbial abundance, diversity, and composition.

Large programs such as the Human Microbiome Project investigate the diversity of the bacterial population associated with the human body, its inter- and intra-personal variability, the influence of endogenous and exogenous factors, and characterizes its principal constituents [**34**].

The intestinal microbiota of infants is very different from the one of adults and shows very important interindividual variability. Similarities appear around 1 year of age and converge towards a more commonly shared adult-like microbiota [**35**].

**Fig. 2:**
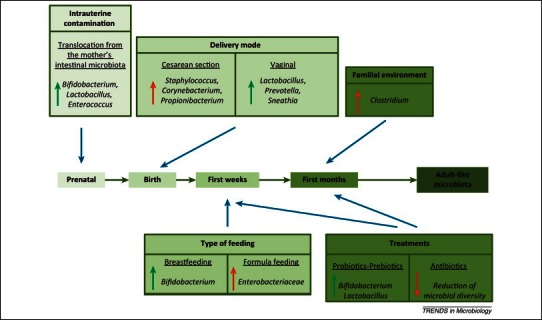
Impact of external factors on the intestinal microbiota of the infant. Green arrows show beneficial modification; red arrows show modification considered negative for a healthy development

The external factors involved in the microbiota change, starting with the prenatal period (intrauterine contamination), continuing from the moment of birth (cesarian section or natural birth), the neonatal period (type of feeding- breastfeeding or formula feeding, tratments), then on sugar (familial environment, treatments) until the adult age, are presented in **Fig. 2** [**36**]. 

**Iatrogenic**

Cranial irradiation or surgery cause hypothalamic damage (see above). Psychotropic medication (e.g., olanzapine and risperidone) and chemotherapeutics (e.g. treatment acute lymphocytic leukemia even without cranial irradiation) have been associated with a greater weight gain in children and adolescents [**37**,**38**].

The possibility of endocrine diseases, congenital and acquired hypothalamic defects, genetic syndromes, and usage of medications affecting the appetite should be considered in the evaluation of the paediatric patient with obesity. However, the vast majority of patients will not have any of these identifiable conditions. Regardless of aetiology, all the patients should be considered for modifiable lifestyle risk factors and screened for the complications of obesity.

## Conclusions

It is important to know the epigenetic stimuli playing a part in the changing of the epi-obesogene gene expression involved in weight homeostasis and energetic balance (adipogenesis, chronic inflammation, appetite, thermogenesis, or the turnover of macronutrients). Obesity and the metabolic syndrome in children originate in the intrauterine life. The role of maternal nutrition during pregnancy is important for the gene expression in the feotus, which will last until adult age and might contribute to the appearance of the metabolic syndrome (HTA, insulin resistance, hyperlipidemia, and abdominal obesity). The knowledge on the connection between obesity and intestinal microbiota allowed the identification of the biomarkers of predisposition to weight gain and the elaboration of strategies for the early treatment. Aiming towards an optimum life style remains the main prophylactic and therapeutic measure in the management of obesity associated with metabolic complications.
